# Time trends in mental well-being: the polarisation of young people’s psychological distress

**DOI:** 10.1007/s00127-017-1419-4

**Published:** 2017-07-11

**Authors:** Andy Ross, Yvonne Kelly, Amanda Sacker

**Affiliations:** Research Department of Epidemiology and Public Health, ESRC International Centre for Lifecourse Studies in Society and Health, University College London, 1-19 Torrington Place, London, WC1E 6BT UK

**Keywords:** GHQ-12, Time trends, Young people, Polarisation, Individualization

## Abstract

**Purpose:**

Previous research on time trends of young people’s mental health in Britain has produced conflicting findings: evidence for deterioration in mental health during the late 20th century followed by stability and slight improvement during the early 21st century is contrasted with evidence showing continued deterioration. The present study adds to the evidence base by assessing time trends in means, variances, and both low and high psychological distress scores covering a similar period.

**Methods:**

GHQ-12 (Likert scale) was regressed on time (adjusting for age) using a sample of young people aged 16–24 between 1991 and 2008 from the British Household Panel Study. Change in variance was assessed using Levene’s homogeneity of variance test across 9-year intervals. Polarisation was assessed by a comparison of the prevalence of scores ≥1 standard deviation and ≥1.5 standard deviations above and below the pooled mean.

**Results:**

There was a small but significant increase in mean GHQ-12 among young women (*b* 0.048; 95% CI 0.016, 0.080) only. Variance increased significantly (*p* < 0.05) across 9-year intervals in seven out of nine comparisons for women and in six out of nine comparisons for men. There were significant increases in low (OR: 1.19; 95% CI 1.05, 1.35), high (OR: 1.27; 95% CI 1.13, 1.42), and very high scores (OR: 1.42; 95% CI 1.23, 1.64) for young women, and increases in low (OR: 1.39; 95% CI 1.21, 1.59) and very low (OR: 1.53; 95% CI 1.21, 1.92) scores for young men.

**Conclusions:**

The evidence suggests a polarisation of the psychological distress of young women in Britain between 1991 and 2008.

## Introduction

In 1995, a seminal review examining contemporary evidence on time trends in psychosocial disorders among youth concluded that it was “clear (there had) been substantial increases in the psychosocial disorders of youth since the Second World War in nearly all developed countries” [1 p. 771]. Increases in youth offending, alcohol and illicit drug use, depression, suicide and suicidal behaviour, and probable increases for eating disorders were demonstrated. Whilst the extent of the evidence was compelling, there were significant limitations regarding its quality [2], a point duly acknowledged by the authors.

Since its publication, there has been a growth in studies examining time trends in emotional and behavioural disorders using data more appropriate for their measurement. More often, this has involved a comparison of the prevalence of disorders across two or more timepoints, measured contemporaneously using identical and validated instruments, often within the context of a social survey.

Results from these studies support those of the original review, showing increases in psychosocial disorders among young people during the latter part of the 20th century [2–7]. Towards the very end of the century, the pattern is more mixed, however: some studies suggest that a plateau was reached or that trends had begun to reverse [8, 9], whilst others claim that these upward trends continued into the early 21st century [10–15]. Results also varied depending on the age of the respondent, disorder examined, and both the instrument and informant used to measure it. For example, parent or teacher reports were more likely to suggest stable or declining trends [8, 9, 16, 17], whereas self-reports tended to suggest increasing problems, particularly internalising, over time [5, 6, 10–15]. Not all findings fit with this pattern [2, 4, 13, 18], a notable exception being studies using the youth report Strengths and Difficulties Questionnaire (SDQ) [9, 19].

In the UK, two research programmes set the tone of the debate. Collishaw and colleagues identified an increase in non-aggressive conduct problems between 1974 and 1999 and in emotional problems between 1986 and 1999 among 15–16 years [2]. However, between 1999 and 2004, these trends either plateaued or declined slightly [9]. In a separate study, they demonstrate an increase in self-reported psychological distress, and self- and parent-rated emotional problems for girls, and in parent-rated emotional problems for boys between 1986 and 2006 [4]. The timing of data collection raises uncertainty about a potential unobserved time trend change in the intervening period, however. In contrast, Sweeting and colleagues identified increases in psychological distress among 15-year-old girls between 1987 and 1999 [6] and among both girls and boys between 1999 and 2006 [12], suggesting that the deterioration of young people’s mental well-being continued into the early 21st century.

The present study adds to the evidence base by examining time trends in psychological distress in young people between 1991 and 2008. It will also attempt to address another criticism of Rutter and Smith’s original review that has received far less attention in the literature.

Fergusson argued that evidence of increasing diversity in spheres assumed to impinge on child development (such as the family, education, and morality) presented in Rutter and Smith [1], was likely to lead to increasing variability in adolescent adjustment, and not simply its increasing pathology [20]. Thus, we should expect to see larger numbers of young people showing positive and prosocial adjustment in addition to increases in those presenting with maladjustment.

Some studies have examined increases in variability in young people’s adjustment [3, 16] and the polarisation of young people’s adjustment over time [15, 18]. An increase in variability in self-esteem and anti-social behaviour was shown among 15-year-old Swedish girls between 1970 and 1996 [18]. This increase took the form of a polarisation of scores in anti-social behaviour and a similar (non-significant) trend for self-esteem. An increase in the variance of depression scores was also found among 16–17-year-old Nordic girls between 1992 and 2010 and among boys between 1992 and 2002 [15]. The prevalence of high depression scores (greater risk) increased for girls and boys between 1992 and 2002, and zero scores (low risk) increased for girls between 1992 and 2010 and for boys between 1992 and 2002, suggesting some polarisation. Studies examining trends in variance alone have demonstrated an increased variance in competency and problem scores among 7–16 years in the USA between 1976 and 1989 [3], but a decreased variance in emotional and behavioural scores among 8 years between 1999 and 2008 in the UK [16].

Rutter and Smith proposed a range of hypotheses for explaining the trends [1], some which were examined in the studies that followed [21–26]. Increased divorce rates, and an increased prevalence of single parent or reconstituted families, were found to be unimportant [2, 9, 22]. Proximal family factors, including parenting behaviours and family relationships (the mechanisms through which divorce or family type might impact adjustment), had also improved over time and had been shown to have had a protective effect on adolescent mental health [21]. Sweeting et al. [25] found an increase in parent–child arguments associated with adolescent emotional problems, but could not discount the possibility of reverse causality.

Changes relating to socio-economic position, including increasing inequality, had not contributed to trends in conduct problems [22], but the evidence relating to emotional problems was more mixed. Increases in psychological distress were shown to be mainly a middle class, female phenomenon [6]. Yet, an income differential in emotional problems has opened up which was attributed to increasing numbers living in rented accommodation and the associated exposure to adverse events, maternal distress, and family dysfunction [23]. There is also evidence that an increased emphasis on educational achievement (or performance) is contributing to increasing psychological distress among adolescents, particularly girls [6, 25].

The present study will examine time trends in psychological distress in 16–24 years in GB to assess hypotheses that population means and the variability of scores have increased over the latter 20th and early 21st centuries. Evidence of increasing variability will be assessed in terms of increasing standard deviation scores over time, and any increase identified subsequently examined to see whether this takes the form of increasing polarisation. We acknowledge that 16–24 is at the upper end of the period of youth covered by Rutter and Smith [1] and older than the majority of subsequent studies. ‘Late youth’ [26] or ‘emerging adulthood’ [27] is, however, an arguably critical period in which young people transition from childhood to early adulthood or from dependency to the beginnings of independency.

## Methods

### Data

Data come from the British Household Panel Survey (BHPS), a multi-purpose panel study of British households carried out annually from 1991 to 2008. The initial 1991 sample consisted of an equal-probability clustered sample of 8167 addresses drawn from the Postcode Address File (PAF), with a subsequent partial household response rate of 74% after adjustment for vacant/non-residential/foreign addresses and the inclusion of multi-occupancy households. The achieved sample comprised 10,300 individuals living in 5500 private dwellings drawn from 250 areas and is representative of the non-institutionalised population of Great Britain [28]. Attrition was highest during the first 5 years of the study with year-on-year attrition down from 14% (1991–2) to 5% (1994–5), remaining approx. 3% thereon (1995–2004) [29]. Data are weighted to produce unbiased parameter estimates of the GB population for that year using the supplied cross-sectional weights that correct for sample design and non-response (including attrition) and allow for new entrants [30]. For further detail on the construction of weights, see Brice et al. [28 pp. A5–1 to A5–12].

The study sample comprises all original sample members (i.e., all members of the households sampled in 1991, including children who became eligible for the adult survey when they were age 16) aged between 16 and 24 in each year from 1991 to 2008, representing 6212 unique individuals who contributed 1–9 years of data (mean 3.7 years), totalling 23,374 person-years of data. Table 2 in the appendix provides sample sizes and for each year non-responders (attrited and age-ineligible) and new sample members (newly eligible and other reasons). Weights were further calibrated to ensure a consistent age distribution of the sample across time.

### Measurement

Psychological distress was measured using the 12-item General Health Questionnaire (GHQ-12), which records the presence and frequency of a range of symptoms, developed as a screening instrument for psychiatric morbidity [31]. Using paper self-completion, respondents are asked whether they have experienced a symptom ‘not at all’, ‘no more than usual’, ‘rather more than usual’ or ‘much more than usual’ in the last few weeks, or for positive worded items, whether their experience was ‘better/more than usual’, ‘same as usual’, ‘less than usual’, or ‘much less than usual’. Negative symptoms include, for example, lost sleep over worry and thinking of self as worthless. Positive symptoms include able to face up to problems and feeling reasonably happy. The GHQ-12 has been validated across many populations, including young people (Cronbach *α* 0.83 [32]; correlation with Present State Examination: *r* = 0.53 [33]).

A number of approaches exist for coding and deriving an overall scale [34]. The Likert scoring method was used (the items are summed giving an overall scale of 0–36) because of its relatively normal distribution and ability to capture variability at both ends of the distribution (low and high levels of psychological distress). The GHQ is one of the commonly used instruments in the assessment of time trends in young people’s adjustment [4–6, 12].

### Data analysis

First, linear regression was used to estimate time trends in mean GHQ-12 scores separately for young men and women, clustering on the individual to resolve an autocorrelation problem associated with the survey’s longitudinal design [each member typically contributes to more than one annual estimate (maximum of nine) for the period in which they were aged 16–24 and remained in the survey]. Stratification by gender was supported by preliminary analysis demonstrating gender-specific trends (*p* < 0.004).

To test the polarisation hypothesis, we used the same two step strategy employed by Wångby et al. [18]. First, Levene’s homogeneity of variances test [35] assessed whether there had been an increase in variance. Where an increase was evident, the tails of the distribution were examined to see whether this was in the form of a polarisation of GHQ-12 scores over time. Again, to resolve the autocorrelation problem, comparisons of the standard deviations were conducted across 9-year interval to ensure independent samples, beginning with a comparison of scores from 1991 and 2000, and ending with a comparison of scores from 1999 and 2008.^1^ To investigate prevalence of scores in the tails of the distribution over time, two separate cutoffs were used: scores at 1 standard deviation or more above and below gender-specific pooled mean scores, and scores at 1.5 standard deviations or more, enabling the examination of the polarisation of scores at a lesser or greater distance from the overall mean. Simultaneous increases in the prevalence of scores in both tails would support the polarisation hypothesis.

In a final step, an average difference in prevalence was estimated for scores at (1) 1 standard deviation or more above the pooled mean; (2) 1 standard deviation or more below the pooled mean; (3) 1.5 standard deviations or more above the pooled mean; and (4) 1.5 standard deviations or more below the pooled mean using a method devised for meta-analysis. The strategy enables assessment of the overall direction of change or average change over the period and is especially useful where the prevalence is particularly low (e.g., at or below 1.5 standard deviations). Relative differences in the prevalence of high and low scores over time were estimated as odds ratios before being analysed using the metan command in STATA S.E 14.1. The analysis was carried out separately for men and women.

## Results

**Fig. 1:**
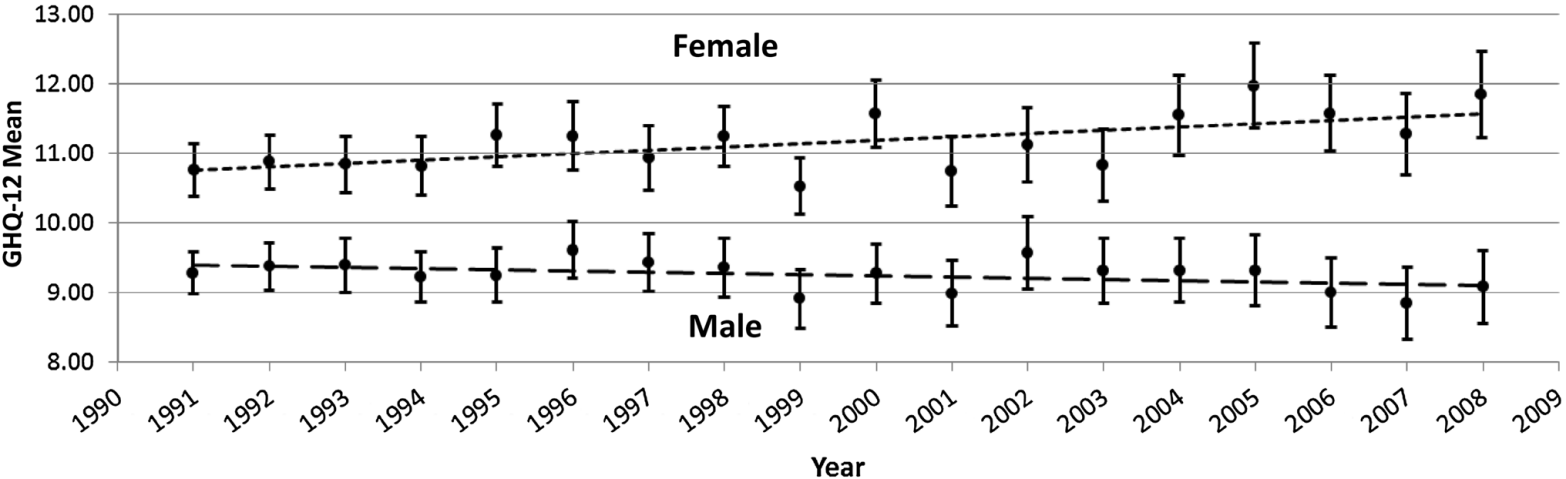
Age-adjusted GHQ-12 mean scores for 16–24 years stratified by gender including confidence intervals and lines of best fit (BHPS: 1991–2008)

Figure 1 plots the age-adjusted GHQ-12 mean scores and linear line of best fit for young men and women aged 16–24, between 1991 and 2008. The associated regression coefficients are for men, *b* −0.017 (95% CI −0.045, 0.011) and for women, *b* 0.048 (95% CI 0.016, 0.080), a small but statistically significant increase in mean GHQ-12 for women and a non-significant decrease for men. The average in 1991 was 1.5 points higher for women than it was for men reflecting expected gender differences, which became significantly more different over the period [female by time interaction: *b* 0.061 (95% CI 0.020, 0.103)]. A time-squared term which assessed a levelling or trend reversal over time was non-significant for both genders.

**Fig. 2:**
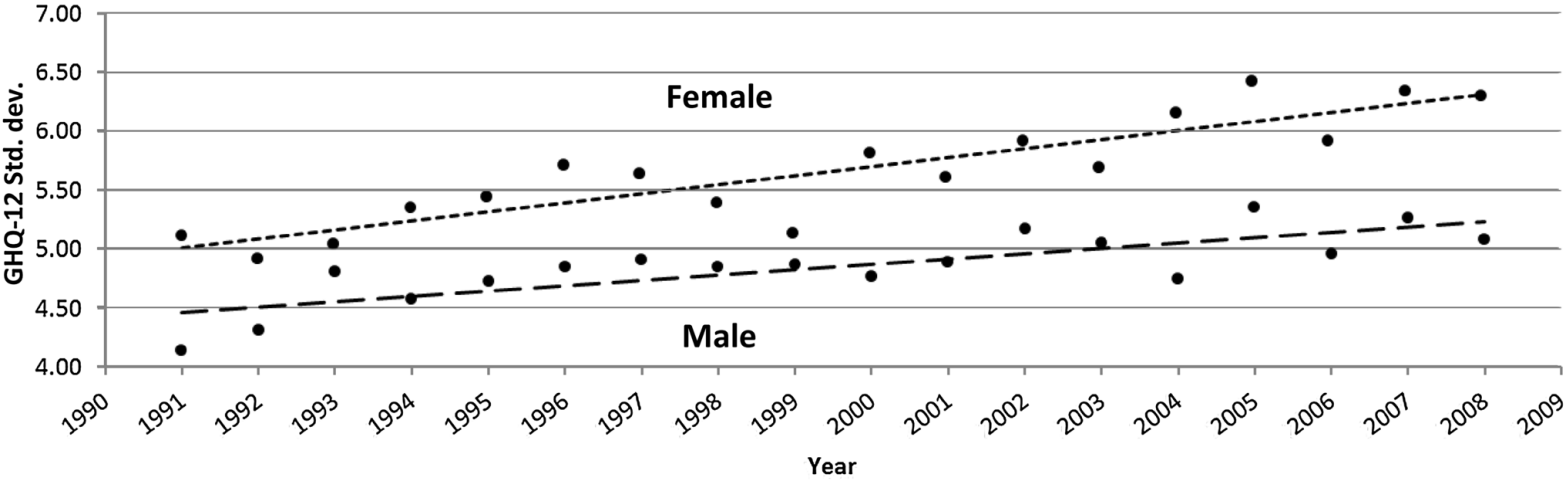
Age-adjusted GHQ-12 standard deviations for 16–24 years stratified by gender including lines of best fit (BHPS: 1991–2008)

Figure 2 plots the aged adjusted standard deviations for GHQ-12 and linear line of best fit from 1991 to 2008 (men: *y* = 4.46 + 0.045*x*; women: *y* = 5.01 + 0.77*x*). Results suggest a relatively linear increase in the variance of GHQ-12 scores over time for both men and women. Furthermore, the standard deviations were higher and the increase a little steeper for young women. By the end of the period, the standard deviation increased from 5.1 to 6.3 for young women and from 4.1 to 5.1 for young men.

Homogeneity of variances was assessed across 9-year intervals beginning with 1991 to 2000 and ending with 1999 to 2008. Results presented in Table 1 show an increase in variance that was statistically significant in six out of nine comparisons for men, and seven out of nine comparisons for women, confirming the trends, as shown in Fig. 1.

**Table 1:** Nine homogeneity of variances tests across 9-year intervals to assess increases in the variance of GHQ-12 scores among 16–24 years between 1991 and 2008 (BHPS)

Comparison *A*–*B*	Men			Women		
	Standard deviation		Ha: ratio <1	Standard deviation		Ha: ratio <1
	*A*	*B*	*p* values	*A*	*B*	*p* values
1991–2000	4.14	4.77	<0.005	5.11	5.82	<0.005
1992–2001	4.31	4.88	<0.005	4.91	5.61	<0.005
1993–2002	4.80	5.17	0.04	5.03	5.91	<0.005
1994–2003	4.58	5.05	0.01	5.34	5.69	0.07
1995–2004	4.72	4.75	0.44	5.44	6.16	<0.005
1996–2005	4.85	5.35	0.01	5.70	6.42	<0.005
1997–2006	4.91	4.96	0.40	5.64	5.91	0.13
1998–2007	4.85	5.27	0.03	5.39	6.34	<0.005
1999–2008	4.87	5.08	0.16	5.12	6.30	<0.005

**Fig. 3:**
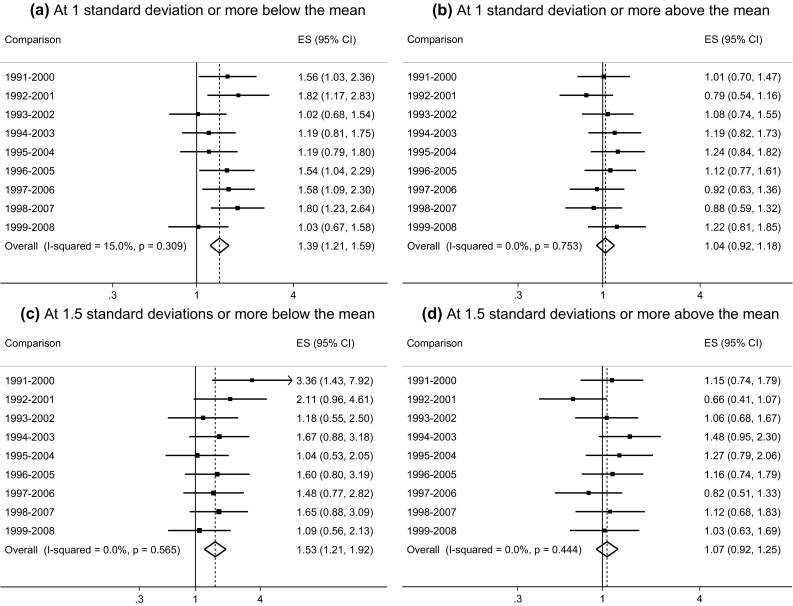
Meta-analysis of odds ratios comparing the proportion of young men at 1 standard deviation and 1.5 standard deviations or move above and below the pooled GHQ-12 mean score across 9-year intervals (BHPS: 1991–2008)

**Fig. 4:**
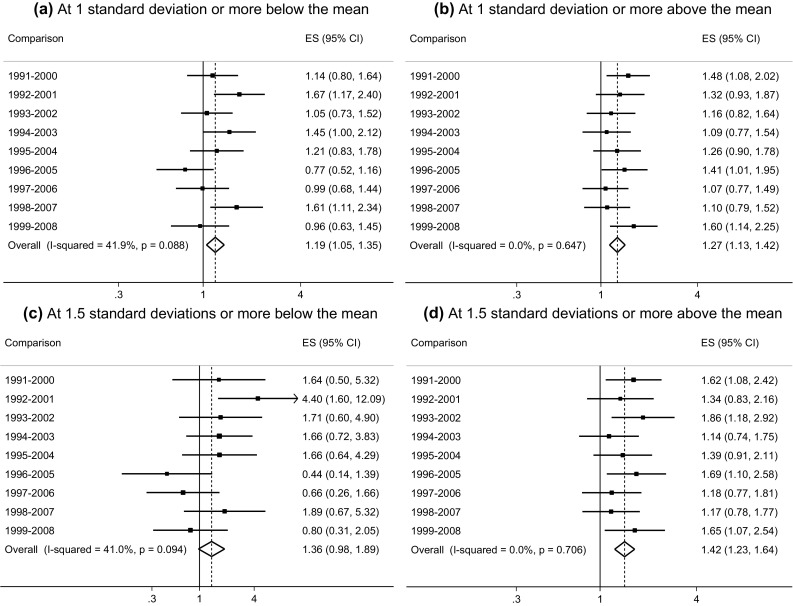
Meta-analysis of odds ratios comparing the proportion of young women at 1 standard deviation and 1.5 standard deviations or move above and below the pooled GHQ-12 mean score across 9-year intervals (BHPS: 1991–2008)

To assess whether this increase took the form of the polarisation of GHQ-12 scores, differences in the prevalence of scores at 1 standard deviation or more (and 1.5 standard deviations or more) above/below the pooled mean were also assessed across 9-year intervals and an ‘average change’ estimated using meta-analysis. Figure 3a–d presents the results for men and Fig. 4a–d presents the results for women.

The average 9-year increase in scores at 1 standard deviation or more below the pooled mean among young men was positive and statistically significant (OR: 1.39; 95% CI 1.21, 1.59) (Fig. 3a). The average increase in scores at 1 standard deviation or more above the pooled mean, although also positive was non-significant (OR: 1.04; 95% CI 0.92, 1.18) (Fig. 3b). Equivalent odds ratios for the average increase in scores at 1.5 standard deviations or more from the mean were OR: 1.53 (95% CI 1.21, 1.92) for scores below the pooled mean and OR: 1.07 (95% CI 0.92, 1.25) for scores above the pooled mean (Fig. 3c, d respectively). Although the increased prevalence in scores above the mean were non-significant, they were nevertheless in the direction hypothesised. Sensitivity analyses assessing increases in the prevalence of scores ≥2 standard deviations (approximately 7% of young men) found a borderline significant increase over time (OR: 1.18; 95% CI 0.98 1.43).

For young women, the average 9-year increase in scores at 1 standard deviation or more below the pooled mean was small but positive (OR: 1.19; 95% CI 1.05, 1.35) and a little larger and positive at 1 standard deviation or more above the mean (OR: 1.27; 95% CI 1.13, 1.42) (Fig. 4a, b). The average increase in scores at 1.5 standard deviations or more below the pooled mean, whilst positive was borderline significant (OR: 1.36; 95% CI 0.98,1.89), however the average increase in scores at 1.5 standard deviations or more above the mean was positive and larger still (OR: 1.42; 95% CI 1.23, 1.64) (Fig. 4c, d respectively). The results therefore support the polarisation of psychological distress among young women.

A concern with a study that uses panel data to assess population trends in psychological distress is the potential impact of differential attrition. A limited attrition analysis carried out to assess its potential impact on our conclusions found that differential attrition related to age was entirely adjusted for and made non-significant for gender using the appropriate weights. A gender differential in drop-out associated with extreme GHQ-12 scores remained, however (results not shown). The odds ratio of missing a subsequent wave was 25% higher among young men with GHQ-12 scores ≥1 standard deviation above the pooled mean and 30% higher for scores ≥1.5 standard deviations. There was no association with GHQ-12 scores and attrition among young women. As a consequence, we are likely to underestimate increases in high levels of psychological distress in young males over time, and reported gender differences may therefore be overstated.

## Discussion

This paper examined time trends in psychological distress among young people in GB between 1991 and 2008 assessing two separate but non-conflicting hypotheses and found evidence supporting both. First, the results show a statistically significant although substantively small increase in mean psychological distress scores of young women, but not men. This adds to findings elsewhere [6, 12] which found a significantly greater increase in the psychological distress of girls, although the sample was younger and the extent of change in these earlier studies was greater.

Second, results for young men demonstrate a clear and consistent increase in low and very low scores over the period, suggesting an overall improvement in levels of psychological distress among young men. There was some indication, however, to suggest an increase in very high scores—scores ≥9.5 points above the overall average on the 0–36 GHQ scale. The increase in low scores alone is nevertheless interesting given that we identified no trend in mean scores for men, suggesting that examining mean differences might otherwise mask trends.

The results lend clear support to the hypothesis of the polarisation of psychological distress in young women. Results show a clear and consistent increase in high and very high scores, confirming the results of the analysis of GHQ-12 mean scores. What this approached had masked and is evident here, however, was a parallel increase in the prevalence of low scores.

Our evidence for the polarisation of young women’s psychological distress adds to the findings of von Soest, Wichstrom [15] and Wångby et al. [18], and in terms of their increasing variance, also those of Achenbach et al. [3]. The results of these earlier studies are a little more equivocal, however. For example, Wångby et al. [18] found no evidence of a change in emotional problems between cohorts in terms of the nine adjustment scales examined, and Sellers et al. [16] also found that variance in emotional and behavioural disorders had decreased over time. Their samples were younger in age than ours, however, and it is plausible that differences in the measures used, the nationality of the sample, as well as the period over which the study carried out may also have contributed to disparities in findings.

Whilst an assessment of the possible cause for these trends is beyond the scope of the present study, we conducted some post hoc descriptive analyses exploring the characteristics of those in the tails of the GHQ-12 distribution, both overall and over time, providing some interesting insights into the underlying patterns of change. Contrary to Langton et al. [23], we found no evidence of an increasing income differential in psychological distress over time, suggesting that the polarisation was not the result of increasing levels of inequality. Instead, we found that young people in the top income quintile were less likely to have high GHQ-12 scores and (among young men) more likely to have low GHQ-12 scores at the beginning of the period examined.

We did, however, find support for Sweeting et al.’s view that an increased emphasis on educational achievement is contributing to increasing psychological distress among girls [6, 25]. Young people with no educational qualifications were more likely to have high GHQ-12 scores. Over time, however, the increase in the prevalence of high GHQ-12 scores for young women was greater among those with higher levels of education, especially degree level qualifications. We considered both income and education in a limited analysis of differential attrition, but found nothing that would undermine findings reported here (results not shown).

Whilst significant inroads have been made on the question of cause, one line of inquiry reported in Rutter and Smith [1] remains significantly under-researched: a proposed cultural shift towards individualistic values, an increased emphasis on self-realisation and fulfilment, and subsequent rise in expectations [36]. It is possible to locate these changes within the wider framework of individualization theory [37], which describes a decline in tradition as the primary source for defining individual lives and identities: a decline in the influence of institutions such as the church and the family, but also including social categories such as gender, social class, and ethnicity. Instead, individuals are assumed to play a more active and central role in defining their own lives and identities. As a consequence, individuals are assumed to take far greater responsibility for their own successes and failures, including factors previously considered personal misfortune, such as structural unemployment, illness, and addiction [37]. Furlong [38] suggests that the perception of personal agency has increased as a consequence, whilst perceptions of social structure have become diminished or obscured, overstating the former. This discrepancy has the effect of raising expectations and aspirations which are then difficult to realise, which can lead to frustration and disappointment, and perhaps ultimately self-blame for that failure [38].

On the other hand, an increase in personal agency, even if in perception alone, has the potential to empower the individual when things go well, in turn bolstering their confidence and self-esteem. Côté proposed a number of diverse pathways of identity formation under the conditions of individualization that have different consequences for young people’s mental health [39]. Accordingly, those with appropriate personal and social resources are better placed to capitalise upon the possibilities that individualization offers, protecting against psychological distress, whilst those without these resources may be at greater risk than hitherto.

Our evidence for the polarisation of young women’s mental well-being over time might, therefore, represent a first step in linking time trends to individualization. Furthermore, the link between increasing psychological distress and an increased emphasis on educational achievement among girls [6, 25] might also be understood within the context of increasing individualization, as might their increasingly higher levels of psychological distress. Some young women may be driven to achieve academically as a consequence of a greater emphasis on personal responsibility for one’s own success or failure, experiencing greater levels of anxiety as consequence. A disparity between perceived personal agency and social structure that continues to frustrate self-realisation is also particularly relevant to the experiences of women. Beck argued, for example, that “the equalisation of men and women cannot be created in an institutional family structure which presupposes their inequality” [37 p. 202]. Continuing gender inequalities (some legislative, some cultural), which serve to frustrate women’s dreams of self-realisation might help explain the observed gender differences in psychological distress over time, though this remains to be tested empirically.

### Strengths and limitations

This study’s use of the BHPS to assess time trends in psychological distress among 16–24 years between 1991 and 2008 has a number of strengths. As a panel study, the format of the survey remains similar between waves of data collection, reducing the risk of changes in question ordering which have been shown previously to impact response [40]. Unlike the majority of studies that use a limited number of data points with which to assess trends over time (typically two or three), BHPS provides 18 data points over the time period under investigation, meaning that estimates are less susceptible to random periodic fluctuations. For example, a simple comparison of GHQ-12 mean scores among women between 1991 and 2001 would have wrongly concluded that psychological distress remained stable (see Fig. 1). It also covers a period in which time trends in British young people’s adjustment are contested. Our findings add to those of Sweeting et al. [12] suggesting that the psychological health of young women (at least) has continued to deteriorate into the early 21st century, although rates of decline are smaller than those found among 15 years in Glasgow and are applicable to Britain more broadly. Our study also goes further than most assessing evidence for the polarisation of young people’s psychological distress.

Whilst significant steps were made to resolve the issue of autocorrelation, it is possible that this has impacted our results. For example, our meta-analyses, which include repeat observations, partially violate the independence assumption, although this was shown to have a limited impact in simulation studies [41]. In addition, the exchangeability assumption that is routinely violated in meta-analyses but which is met here, has been shown to matter more [42]. Restrictions of software mean that we can only account for one level of clustering, which in this case was time within individual. As a household panel study, our data are also clustered at the household level with intraclass correlations of 0.16 and 0.11 for males and females, respectively. However, an exploratory analysis in which household clustering was considered found only fractional increased confidence intervals, supporting the findings of a more thorough assessment of ignoring person-group clustering in the BHPS [43]. A linked concern is that respondents may replicate their answers to the GHQ-12 over the duration of the panel. Despite this, a separate study found no evidence of retest effects associated with GHQ-12 in the BHPS [44]. Bias associated with sample attrition is corrected for using probability weights, and an analysis of differential attrition suggests that it has only a limited impact on our conclusions. We may underestimate increases in high levels of psychological distress in the young male population over time, and our identified gender differences might therefore be overstated. Furthermore, the risk remains that residual bias could lead to further underestimation of the levels of psychological distress. The age of our sample prevents direct comparison with the majority of previous studies, which tended to measure mental health during the mid-teenage years. Finally, it should also be noted that whilst the last data point was in 2008, this was at the very beginning of the Great Recession, and we are unable to capture the impact of this global event on young people’s psychological distress.

## Conclusion

An assessment of mean GHQ-12 scores among British 16–24 years between 1991 and 2008 found a small but significant increase in psychological distress among young women, but not men. The variability of GHQ-12 scores increased for both genders, although standard deviations were higher and the increase a little steeper for young women. This increase in variability took the form of a clear polarisation of psychological distress among young women and the possibility of polarisation in young men once differential attrition is considered. For young women a general increase in psychological distress was found, together with evidence of a growing minority with much better mental well-being. This study underlines the importance of assessing the variability of adjustment scores over time as well as their average.

## Appendix

See Table 2.

**Table 2:** Sample sizes, non-response, and new sample members [unweighted *n* (%)]

	Survey year																	
	1991	1992	1993	1994	1995	1996	1997	1998	1999	2000	2001	2002	2003	2004	2005	2006	2007	2008
	*n* (%)	*n* (%)	*n* (%)	*n* (%)	*n* (%)	*n* (%)	*n* (%)	*n* (%)	*n* (%)	*n* (%)	*n* (%)	*n* (%)	*n* (%)	*n* (%)	*n* (%)	*n* (%)	*n* (%)	*n* (%)
Previous wave sample^a^																		
Returners	n/a	1098 (72.7)	1060 (70.2)	1088 (73.7)	1066 (71.7)	1077 (74.6)	1127 (74.4)	1080 (72.7)	1035 (76.7)	965 (74.0)	941 (74.4)	909 (72.7)	858 (74.0)	845 (73.7)	822 (74.7)	867 (78.4)	860 (76.5)	820 (74.3)
Non-response (true)	n/a	248 (16.4)	282 (18.7)	233 (15.8)	258 (17.4)	195 (13.5)	215 (14.2)	242 (16.3)	193 (14.1)	184 (14.1)	189 (14.9)	206 (16.5)	177 (15.3)	173 (15.1)	140 (12.7)	132 (11.9)	170 (15.1)	165 (15.0)
Non-response (turned 25)	n/a	248 (10.9)	282 (11.1)	156 (10.6)	163 (11.0)	171 (11.9)	173 (11.4)	164 (11.0)	140 (10.2)	155 (11.9)	135 (10.7)	136 (10.9)	125 (10.8)	128 (11.2)	139 (12.6)	107 (9.7)	94 (8.4)	119 (10.8)
Current wave sample^b^																		
Returners	n/a	1098 (72.7)	1060 (71.8)	1088 (73.2)	1066 (73.9)	1077 (71.1)	1127 (75.8)	1080 (79.0)	1035 (79.4)	965 (76.3)	941 (75.2)	909 (78.4)	858 (74.9)	845 (76.8)	822 (74.3)	867 (77.1)	860 (77.9)	820 (80.7)
New sample (turned 16)	n/a	144 (17.8)	173 (18.4)	265 (17.8)	240 (16.6)	271 (17.9)	219 (14.7)	165 (12.1)	130 (10.0)	169 (13.4)	176 (14.1)	125 (10.8)	148 (12.9)	122 (11.1)	148 (13.4)	125 (11.1)	88 (8.0)	99 (9.7)
New sample (other)	n/a	268 (9.5)	181 (9.9)	134 (9.0)	137 (9.5)	167 (11.0)	140 (9.4)	123 (9.0)	139 (10.7)	131 (10.4)	134 (10.7)	126 (10.9)	140 (12.2)	134 (12.2)	136 (12.3)	132 (11.7)	156 (14.1)	97 (9.6)
Total *n*	1511	1510	1477	1487	1443	1515	1486	1368	1304	1265	1251	1160	1146	1101	1106	1124	1104	1016

^a^Showing for each year previous respondents who remained in the study (returners), non-response as a consequence of attrition (true), and of turning age 25 (turned 25)

^b^Showing for each year previous respondents who remained in the study (returners), new sample members as a consequence of turning 16 (turned 16), and for other reasons such as their being available for interview in the current but not previous wave of data collection (other)

## Footnotes

1 Closer time intervals could include repeated observations from the same individuals.
